# Intravenous Steroids Do Not Improve Short-Term Outcomes of Patients With Crohn’s Disease Presenting With an Acute Small Bowel Obstruction

**DOI:** 10.1093/crocol/otae064

**Published:** 2025-01-08

**Authors:** Mariely Garcia, Anketse Debebe, Farhan Mahmood, Sharon Nirenberg, Alexa Rendon, Eunyoung Yang, Jiani Xiang, Jean-Frédéric Colombel, Tamara Kahan, Ghoncheh Ghiasian, Adam S Faye, Irving Levine, Michael Farber, Michael Ramada, Tisor Omoakhe, Keith Sultan, David B Sachar

**Affiliations:** Icahn School of Medicine at Mount Sinai, New York, NY, USA; Department of Genetics and Genomic Sciences, Icahn School of Medicine at Mount Sinai, New York, NY, USA; Icahn School of Medicine at Mount Sinai, New York, NY, USA; Department of Scientific Computing, Icahn School of Medicine at Mount Sinai, New York, NY, USA; Department of Genetics and Genomic Sciences, Icahn School of Medicine at Mount Sinai, New York, NY, USA; Icahn School of Medicine at Mount Sinai, New York, NY, USA; Icahn School of Medicine at Mount Sinai, New York, NY, USA; The Dr. Henry D. Janowitz Division of Gastroenterology, Icahn School of Medicine, New York, NY, USA; NYU Langone Health, NYU Grossman School of Medicine, New York, NY, USA; NYU Langone Health, NYU Grossman School of Medicine, New York, NY, USA; NYU Langone Health, NYU Grossman School of Medicine, New York, NY, USA; Department of Medicine, Northwell Health, Manhasset, NY, USA; Department of Medicine, Northwell Health, Manhasset, NY, USA; College of Osteopathic Medicine, New York Institute of Technology, Old Westbury, NY, USA; Department of Medicine, Northwell Health, Manhasset, NY, USA; Department of Medicine, Northwell Health, Manhasset, NY, USA; Department of Medicine, Division of Gastroenterology, Northwell Health, Manhasset, NY, USA; The Dr. Henry D. Janowitz Division of Gastroenterology, Icahn School of Medicine, New York, NY, USA

**Keywords:** Crohn’s disease, small bowel obstruction, medical management, corticosteroids, strictures

## Abstract

**Background:**

Intravenous (IV) steroids are commonly used to treat acute flares of Crohn’s disease (CD). However, it is unclear if they are beneficial in the setting of uncomplicated small bowel obstruction (SBO). We sought to examine if IV steroid administration improved short-term outcomes in patients with CD hospitalized for acute, uncomplicated SBO across three New York City hospital systems.

**Methods:**

This retrospective study included patients ≥ 18 years old admitted between January 1, 2011, and December 31, 2019, with Crohn’s disease and an admission diagnosis of uncomplicated acute SBO, defined as cases without adhesions, fistula, phlegmon, and sepsis. Primary endpoints (length of stay and frequency of surgery) were compared between patients who received IV steroids upon admission and those who did not.

**Results:**

Our analysis included 674 unique patients. Ninety-two (14%) received IV steroids, and 582 (86%) did not. IV steroid use did not result in shorter hospital stays (median days [IQR]: 3.0 (2.0-5.5) days vs 3.0 (2.0-6.0) days in the no-steroid group, *P* = .65) or reduce the need for surgery (4 patients (4.4%) vs 28 patients (4.8%) in the no-steroid group, *P* = .85). Sex, age, disease duration, concomitant biologic therapy, and NG tube placement did not independently contribute to either outcome.

**Conclusions:**

These findings suggest that IV steroid administration for uncomplicated SBO in CD patients does not decrease hospital length of stay or need for surgery. Further research may help identify specific obstruction patterns or other therapies associated with different outcomes.

## Graphical Abstract

**Graphical Abstract:**
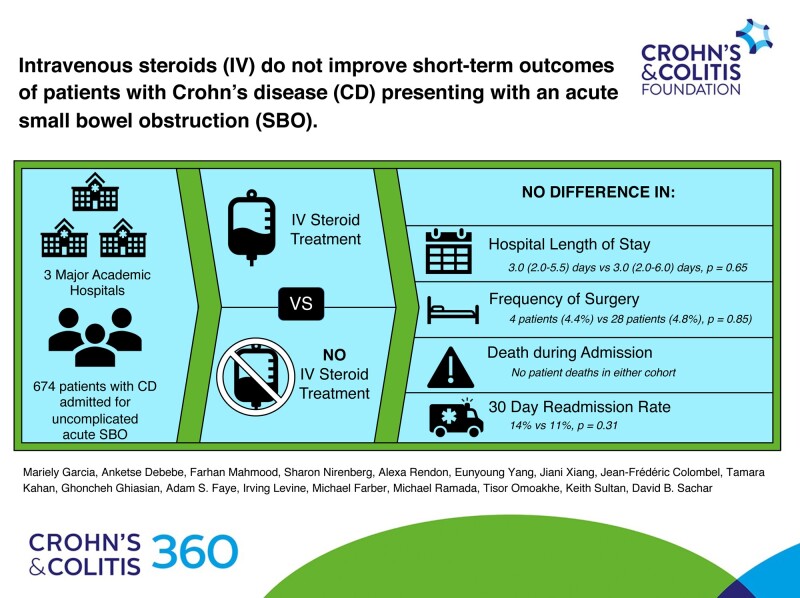

## Introduction

Crohn’s disease (CD) is a type of inflammatory bowel disease that causes inflammation of the digestive tract, often leading to abdominal pain, severe diarrhea, fatigue, weight loss, and malnutrition. The incidence of CD has been increasing globally and in North America.^1,2^

Most societies recommend that initial treatment of “severe, clinically active” CD be conservative and include bowel rest with parenteral nutritional support, hydration, electrolyte replacement, and systemic administration of corticosteroids.^3–6^ Notably, “severe, clinically active” CD can manifest in many ways, including, but not limited to, small bowel obstruction (SBO).^6^

In this respect, clinicians may feel compelled to hasten SBO resolution by supplementing bowel rest and hydration with a brief course of systemic steroids.^7–10^ There is a plausible theoretical rationale for the use of steroid therapy in this context given the inflammatory component of stricture pathophysiology.^3^ However, there is anecdotal evidence to suggest that in the setting of SBO, the administration of systemic corticosteroids has a similar impact as intestinal decompression with nasogastric tube placement alone.^11^ SBO episodes can be frequent and often self-resolve. Considering the adverse effect profile of steroids, limiting their use can be beneficial to both patients and hospital systems.^3^

In the absence of empirical evidence, current guidelines do not make it clear if the recommendation to use steroids should apply to the subset of patients with acute SBO. It is possible that interventions for “severe, clinically active” CD differ in efficacy depending on how an acute flare manifests (eg, SBO alone vs constellation of pain, fever, diarrhea, fatigue). Therefore, we conducted a retrospective study to assess if IV steroid administration at admission improved outcomes for CD patients hospitalized for acute SBO. We chose to focus on SBO without evidence or the possibility of adhesions, fistula, phlegmon, and sepsis, and we refer to this as uncomplicated SBO. Delineating if and when steroids provide benefits for SBO resolution will help reduce waste of resources and limit adverse events associated with steroid use.

## Materials and Methods

### Study Setting

This was a retrospective study conducted across three academic medical institutions in New York City: Mount Sinai Health, Northwell Health, and NYU Langone. A data collaboration agreement was obtained to enable data sharing, and the study protocol and materials were approved by the institutional review boards at each center. All listed authors contributed to the collection and analysis of data as well as the approval of this manuscript.

### Study Population

**Figure 1.**
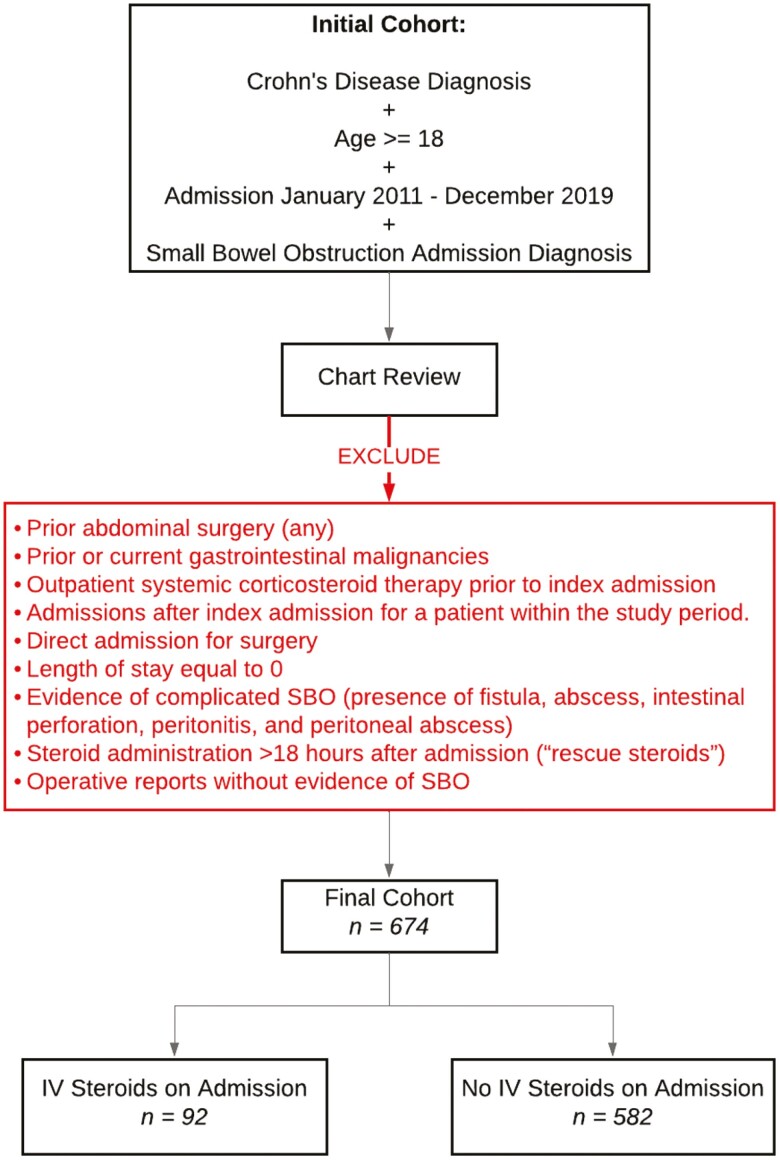
Process for cohort identification.

Patients ≥ 18 years old admitted to our institutions from January 1, 2011, to December 31, 2019, with a diagnosis of Crohn’s disease and an admission diagnosis of acute SBO were included for analysis. The diagnosis of SBO was identified by using ICD-10-CM codes (K56.6*). Quantitative distinctions were not drawn among degrees of obstruction (ie, “partial” vs “complete”); all cases were severe enough to require hospitalization. Patients with prior abdominal surgery were excluded to ensure that all cases listed as acute SBO were due to uncomplicated Crohn’s disease strictures with no possibility of surgical adhesive obstruction (Figure 1). We also excluded cases where steroids were administered 18 or more hours after admission to distinguish between steroids used as a primary therapy vs steroids used as “rescue therapy” in the setting of other failed interventions and/or worsening disease. Patients who were admitted more than once during the study period were only counted once, and the data from their first admission were analyzed.

### Data Collection

Investigators from each participating institution identified patients who met the study inclusion criteria. A manual chart review was done for each case to remove those that met any of our exclusion criteria, namely, those with any prior abdominal surgery, steroid use as an outpatient at the time of admission, no IV steroid administration until after 18 h of admission, and direct admission for planned surgery. The operative reports of those who underwent surgical intervention were reviewed, and those mentioning complications of adhesions, perforation, phlegmon, abscess, fistula, or peritonitis were excluded. The performance of resection or strictureplasty was documented in all surgical cases.

Data regarding demographics (age, sex, duration of Crohn’s disease) and hospital course were collected for the remaining cohort. Disease duration was defined as the difference, in years, between the first documented diagnosis of CD in the electronic medical record and the time of admission. We also recorded nasogastric (NG) tube placement and adalimumab or infliximab (biologics) administration at any point during hospitalization.

### Outcome Measures

Our primary endpoints were the patient length of stay in the hospital and the occurrence of surgery during admission.

### Statistical Analysis

Data analysis included descriptive statistics (days with SD) for continuous variables and percentages for categorical variables. Between-group comparisons were performed using Proc Genmod for continuous variables (LOS) and Proc Logistics for categorical variables (surgery). We used Proc Genmod to test the differences between the steroid vs no-steroid groups and stratified by subgroup (sex, age, duration of disease, NG tube placement, and biologic administration). We used Proc logistics to test the differences between the steroid vs no-steroid groups and also stratified by subgroup (sex, age, duration of disease, NG tube placement, and biologic administration). *P*-values ≤ .05 were considered significant. All analyses were done using excel and SAS software v9.3 software (SAS Institute).

## Results

We identified 674 unique patients with CD who were admitted for an SBO during our study time frame across our 3 institutions. Ninety-two (14%) of those patients received IV steroids at the time of admission, and 582 (86%) did not (Table 1). Abdominal imaging studies (CT and/or MRI) were performed in 83% of all patients (88% of the steroid group and 82% of the no-steroid group). All steroid-treated patients received either hydrocortisone or methylprednisolone intravenously in standard doses. There were 49 (11%) all-cause readmissions within 30 days of discharge among Sinai patients; there was no significant difference in the distribution of these cases between steroid-treated and nonsteroid-treated patients (14% of the steroid group, 11% of the no-steroid group, *P* = .31). Readmission data from Northwell and NYU were not available. There was no significant difference in sex, NG tube placement or concurrent use of biologics between the no-steroid and steroid groups (*P* = .63, *P* = .58, and *P* = .18, respectively). Patients in the steroid group were marginally younger than in the no-steroid group (41.1 [17.7] vs 45.7 [18], respectively) and had a shorter disease duration (4.3 years [7.9] vs 6.8 years [10.8], *P* = .001).

**Table 1.** Comparison of demographics, disease duration, and co-therapies during hospitalization for steroid vs no-steroid groups.

Study group			
Total number	Steroid 92	No-steroid 58	*P*-value
Age—years (SD)	41.1 (17.7)	45.7 (18.0)	.03^a^
Male—*n* (%)	50 (54.4%)	332 (57.0%)	.63
Female—*n* (%)	42 (45.7%)	250 (43.0%)	
Duration of disease—mean (SD)	4.3 (7.9)	6.8 (10.8)	.01^a^
NG tube (Yes)—*n* (%)	38 (41.3%)	223 (38.3%)	.59
Biologic (Yes)—*n* (%)	4 (4.4%)	12 (2.1%)	.18

^a^Denotes statistical significance.

There were no significant differences between the steroid and no-steroid groups for our primary endpoints of hospital LOS (median days [IQR]: 3.0 (2.0-5.5) days vs 3.0 (2.0-6.0) days, respectively; *P* = .65) and frequency of surgery (4 patients (4.4%) vs 28 patients (4.8%), respectively, *P* = .85). There were no patient deaths in any cohort. Logistic regression showed that sex, age, disease duration, concomitant biologic therapy, and NG tube placement did not independently contribute to either outcome measure (surgery, LOS) (Table 2). Results were consistent among the three hospital subgroups (Supplementary Table 1).

**Table 2.** (a) Comparison of hospital length of stay for steroid vs no-steroid groups stratified by subgroups; (b) Comparison of frequency of surgery for steroid vs no-steroid groups.

(a)				
Study group				
Total number	Steroid 92		No- steroid 582	*P*-value
*Median length of stay, days (IQR)*				
All	3 (2, 5.5)		3 (2, 6)	.65
Male	3 (2, 5)		3 (2, 6)	.76
Female	4 (3, 6)		4 (2, 6)	.78
Disease duration 0-2 years	4 (2.5, 7)		4 (2, 7)	.60
Disease duration 2-5 years	3 (2, 4)		3 (2, 5.5)	.82
Disease duration 5 + years	3 (2, 5)		3 (2, 5)	.92
NG tube	4 (3, 5)		4 (3, 7)	.35
No NG tube	3 (2, 7)		3 (2, 5)	.44
Biologic	3.5 (2, 12)		4 (2, 5.5)	.90
No biologic	3 (2, 5.5)		3 (2, 6)	.65
(b)				
*Number of surgeries (%)*				
All	4 (4.4%)	28 (4.8%)		.85
Male	2 (4%)	16 (4.8%)		.80
Female	2 (4.8%)	12 (4.8%)		.99
Disease duration 0-2 years	3 (8.3%)	14 (5.6%)		.46
Disease duration 2-5 years	0 (0%)	5 (4.6%)		1.00
Disease duration 5 + years	1 (2.9%)	6 (4.7%)		1.00
NG tube	2 (5.3%)	10 (4.5%)		.69
No NG tube	2 (3.7%)	18 (5%)		1.00
Biologic	1 (25%)	0 (0%)		.25
No biologic	3 (3.4%)	28 (4.9%)		.79

## Discussion

Our study demonstrates that IV steroid administration for uncomplicated SBO in patients with CD did not significantly reduce hospital length of stay or need for surgery when compared to no IV steroid administration. This finding suggests that the widespread recommendation for steroid use in “severe, clinically active” CD may not apply specifically to cases of uncomplicated SBO.

It is noteworthy that in our study, only a minority of patients were treated with steroids upon admission. This was surprising given current guidelines. However, we speculate that this behavior stems in part from the fact that all three health systems in this study are academic centers with more hands-on experience. Therefore, they may be less prone to reflexive ordering and more cognizant of empirical trends. We also noted that although there was a significant difference in disease durations between groups, there were no differences in outcome measures. There were no patient deaths in any cohort. We did not assess postoperative complications in the steroid vs no-steroid group, but there has been literature documenting worse postoperative outcomes in patients who receive preoperative steroids.^5,8,9,12,13^

SBO is usually the result of both inflammatory and fibromuscular components; medical therapy targets primarily the inflammatory component.^14^ Therefore, many studies have suggested that determining the predominant driver is essential for determining the appropriate therapeutic modality.^10,15–17^ There have been several studies investigating the efficacy of oral and IV steroid use for Crohn’s disease broadly, but to our knowledge, no high-quality clinical trials have looked specifically at steroid efficacy in acute, uncomplicated SBO.^18–22^ However, there have been several investigations into alternative therapies like TNF-alpha inhibitors. The CREOLE study, for example, showed that over 50% of patients treated with adalimumab avoided surgical intervention 4 years after treatment initiation, but there was no control group for comparison.^23^ The rationale is clear for using steroids to reduce the inflammatory components of strictures. Decades of experience indicate that anti-inflammatory medications certainly play a role in overall CD management, but in the setting of acute uncomplicated SBO, steroids may not provide any incremental benefit. Ongoing and future studies will help distinguish which features of SBO make a patient most amenable to steroid or other anti-inflammatory measures.^24^

There are several important limitations in our study. As a retrospective review, the design offers no assurance that the disease severity, phenotypes, and prior histories of the patients in either group were the same. Data gathered from our charts were limited to encounters at our respective institutions and therefore do not include information about medical care received elsewhere. Also, disease duration was determined from dates of diagnosis recorded upon chart review, a crude estimate, at best, of actual disease duration. Moreover, the identification of patients with Crohn’s disease and acute SBO was made based on diagnosis codes, and it is therefore possible that patients were missed. Finally, data on potentially confounding data like smoking status, diet, and all prior medications were not included in this study. Nevertheless, our strict exclusion criteria and the consistency of findings across institutions make us confident that our data and results are generalizable despite the aforementioned limitations. Additionally, our unselected inclusion of all eligible cases and our multivariable analyses of many potentially confounding factors reduced the likelihood of any selection biases.

## Conclusion

While clinical judgment should still be employed in every individual case, this study does not support the routine use of IV steroids in all patients with CD hospitalized for acute uncomplicated SBO.

## Supplementary Material

otae064_suppl_Supplementary_Table_S1

## Data Availability

The data that support the findings of this study are available from the corresponding author upon reasonable request.
