# Supplementary material for: Intravenous Steroids Do Not Improve Short-Term Outcomes of Patients With Crohn’s Disease Presenting With an Acute Small Bowel Obstruction
Source: Crohns Colitis 360. 2025 Jan 8;7(1):otae064. doi: 10.1093/crocol/otae064 (PMC11744190; doi:10.1093/crocol/otae064)
Supplement: otae064_suppl_Supplementary_Table_S1 [file otae064_suppl_supplementary_table_s1.docx]

**Supplementary Table 1:** Comparison of demographics, disease duration, and co-therapies during hospitalization for steroid vs no steroid groups at each hospital.

| *By Institution* |  | Mount Sinai | | | NYU | | | Northwell | | |
| --- | --- | --- | --- | --- | --- | --- | --- | --- | --- | --- |
|  |  | **Steroid** | **No Steroid** | **p-Value** | **Steroid** | **No Steroid** | **p-Value** | **Steroid** | **No Steroid** | **p-Value** |
| Total number |  | 49 | 386 |  | 25 | 27 |  | 18 | 169 |  |
| Age (avg,[SD]) |  | 41 (16.3) | 44.9 (17.2) | 0.29 | 36.3 (19.1) | 35.2 (14.4) | 0.81 | 48.1  (18.0) | 49.1  (20.0) | 0.81 |
| Sex (n,[%]) | Male | 30  (61.2%) | 215 (55.7%) | 0.46 | 11  (44.0%) | 19  (70.4%) | 0.06* | 9  (50.0%) | 98 (57.7%) | 0.52 |
|  | Female | 19  (38.8%) | 171 (44.3%) |  | 14  (56.0%) | 8  (29.6%) |  | 9  (50.0%) | 71 (42.0%) |  |
| Disease duration  (year) (n,[%]) | 0-2 years | 25  (51.0%) | 235 (60.9%) | 0.01* | 11  (44.0%) | 12  (44.4%) | 0.90 | 0  (0.0%) | 4 (2.4%) | 0.19 |
|  | 3-5 years | 10  (20.4%) | 94  (24.4%) |  | 2  (8.0%) | 1  (3.7%) |  | 0  (0.0%) | 13 (7.7%) |  |
|  | 5+ years | 14  (28.6%) | 57  (14.8%) |  | 12  (48.0%) | 14  (51.9%) |  | 9  (50.0%) | 56 (32.9%) |  |
|  | Un-known | 0  (0.0%) | 0  (0.0%) |  | 0  (0.0%) | 0  (0.0%) |  | 9  (50.0%) | 96 (56.8%) |  |
| Nasogastric (NG) tube placement (n,[%]) | Yes | 34  (69.4%) | 265 (68.7%) | 0.92 | 9  (36.0%) | 11  (40.7%) | 0.73 | 7  (38.9%) | 86 (50.9%) | 0.33 |
|  | No | 15  (30.6%) | 121 (31.4%) |  | 16  (64.0%) | 16  (59.3%) |  | 11  (61.1%) | 83 (49.1%) |  |
| Biologic administration (n,[%]) | Yes | 2  (4.1%) | 10  (2.6%) | 0.63 | 2  (8.0%) | 0  (0.0%) | 0.14 | 0  (0.0%) | 2 (1.2%) | 0.64 |
|  | No | 47  (95.9%) | 376 (97.4%) |  | 23  (92.0%) | 27  (100.0%) |  | 18  (100.0%) | 167 (98.8%) |  |

*****Denotes statistical significance
